# The Cap-Independent Translation of Survivin 5′UTR and HIV-1 IRES Sequences Is Inhibited by Oxidative Stress Produced by *H. pylori* Gamma-Glutamyl Transpeptidase Activity

**DOI:** 10.3390/biom16010164

**Published:** 2026-01-19

**Authors:** Mariaignacia Rubilar, Nicolás Carrasco-Véliz, Maritza P. Garrido, María I. Silva, Andrew F. G. Quest, María Fernanda González, Esteban Palacios, Joan Villena, Iván Montenegro, Manuel Valenzuela-Valderrama

**Affiliations:** 1 Laboratorio de Carcinogénesis Molecular, Instituto de Investigación y Doctorados, Facultad de Medicina y Ciencias de la Salud, Universidad Central de Chile, Lord Cochrane 418, Santiago 8320000, Chile; mariaignacia.rubilar.20@gmail.com (M.R.); nicolascarrascoveliz@gmail.com (N.C.-V.); epcontre@gmail.com (E.P.); 2 Laboratorio de Endocrinología y Biología de la Reproducción, Departamento de Obstetricia y Ginecología, Hospital Clínico Universidad de Chile, Avenida Santos Dumont 999, Independencia, Santiago 8380456, Chile; mgarrido@hcuch.cl; 3 Centro de Estudios Avanzados en Enfermedades Crónicas (ACCDiS), Sergio Livingstone 1007, Independencia, Santiago 8380492, Chile; aquest@u.uchile.cl (A.F.G.Q.); mfe.gonzalez@gmail.com (M.F.G.); 4 Laboratorio de Comunicaciones Celulares, Centro de estudios en Ejercicio, Metabolismo y Cáncer (CEMC), Núcleo Interdisciplinario en Biología y Genética, Instituto de Ciencias Biomédicas (ICBM), Facultad de Medicina, Universidad de Chile, Independencia, Santiago 8380000, Chile; 5 Centro Interdisciplinario de Investigación Biomédica e Ingeniería para la Salud (MEDING), Facultad de Medicina, Campus de la Salud, Universidad de Valparaíso, Angamos 655, Viña del Mar 2520000, Chile; juan.villena@uv.cl (J.V.); ivan.montenegro@uv.cl (I.M.)

**Keywords:** IRES, translation, 5′UTR, bicistronic assay, *H. pylori*, Survivin

## Abstract

Background: Survivin is an anti-apoptotic protein highly expressed during embryonic development and, in adults, mainly in the gastrointestinal epithelium. Its levels decrease in human gastric tissue and cultured cells upon exposure to *Helicobacter pylori* gamma-glutamyl transpeptidase (GGT), though the underlying mechanism remains unclear. Objective: We aimed to investigate the role of cap-independent translation driven by the Survivin 5′ untranslated region (5′UTR) in response to *H. pylori* infection in vitro. Methodology: Human cell lines (AGS, GES-1, HeLa, HEK293T) were used alongside bicistronic and monocistronic (Firefly/Renilla luciferases) reporter assays to assess short and long variants of the Survivin 5′UTR and HIV-1 internal ribosome entry site (IRES) sequences. Additional methods included in vitro transcription/translation, RT-qPCR, agarose gel electrophoresis, Western blotting, coupled/uncoupled translation assays, and siRNA silencing. Results: The short variant of the Survivin 5′ UTR supported cap-independent translation, like the HIV-1 IRES. Notably, *H. pylori* infection suppressed this translation in a GGT-dependent manner in gastric cells, and a similar reduction was observed following treatment with ATO, a known prooxidant. Conclusion: The Survivin 5′UTR exhibits cap-independent translation activity that is inhibited by *H. pylori* in a GGT-dependent manner, likely via oxidative stress. This mechanism helps to explain the downregulation of Survivin during gastric infection and indicates that oxidative stress can negatively affect both cellular and viral IRES-mediated translation.

## 1. Introduction

Survivin (BIRC5) is a member of the inhibitor-of-apoptosis protein (IAP) family that is ubiquitously expressed during embryonic and fetal development but generally absent in normal adult tissues, except for certain proliferating stem cell populations [1]. The gastric mucosa represents an exception to this general rule, as Survivin is expressed in normal gastric epithelial cells; however, its loss correlates with mucosal damage associated with chronic *Helicobacter pylori* infection through a mechanism that is not fully understood; but is thought to involve the generation of reactive oxygen species (ROS) mediated by the bacterial gamma-glutamyl transpeptidase (GGT) activity [2,3]. Conversely, Survivin is often upregulated in cancer, where it promotes tumor growth and cell survival. Its expression is inversely correlated with patient prognosis following chemotherapy. Therefore, Survivin is considered both a potential biomarker and a therapeutic target in cancer [4,5,6]. Understanding the mechanisms that regulate Survivin expression is of considerable biological and clinical interest. This is due to its essential role in maintaining gastric epithelial integrity and its frequent overexpression in malignancy.

Survivin’s cytoprotective role is linked to its N-terminal Zn^2+^-binding BIR domain, shared with other IAPs [7]. This domain contributes to caspase inhibition [8], stabilization of XIAP [9], suppression of SMAC/DIABLO [10], and activation of mitotic regulators such as Aurora B, required for chromosomal passenger complex stabilization [11]. Beyond apoptosis inhibition, Survivin modulates pathways important for cancer progression, including angiogenesis and migration [12,13]. Its expression is cell-cycle regulated, peaking in S and G2/M phases [14], and Survivin pre-mRNA undergoes alternative splicing to generate six protein variants [15]. Transcriptional regulation involves NF-κB, STAT3, HIF1α, β-catenin–Tcf-Lef, and p53 [12], while post-translational modifications, such as Thr34 phosphorylation, influence its stability and anti-apoptotic function [16,17,18]. Although transcriptional and post-transcriptional mechanisms are well characterized [12,16,17,19,20,21,22], translational control of Survivin remains understudied.

Canonical cap-dependent translation initiates through recognition of the 5′ cap by the eIF4F complex, recruitment of the 43S pre-initiation complex via eIF4G–eIF3 interaction, and scanning to the start codon [23,24,25]. PABP enhances this process by interacting with eIF4G and bridging the poly(A) tail to the cap-binding machinery [26]. Global translation is regulated through phosphorylation of eIF2α, inhibition of eIF4E–eIF4G interaction by 4E-BPs, or cleavage of translation factors such as eIF4G and PABP [27].

Alternatively, mRNA-specific control—where a group of defined mRNAs is regulated without affecting general protein biosynthesis—relies on regulatory proteins and RNA-binding factors that recognize specific primary or secondary structures present in the 5′- or 3′-untranslated terminal regions (UTRs) of the target mRNAs [24,25]. Secondary structures in 5′UTRs also strongly influence initiation, often limiting ribosomal recruitment and scanning efficiency [25,28], although in some cases they facilitate cap-independent initiation by stabilizing RNA–protein interactions [29]. Internal ribosome entry sites (IRES), initially identified in viral RNAs such as HIV-1 and EMCV, have also been described in several cellular mRNAs—including XIAP, Cyclin D1, p53, BAX, and c-Myc—providing alternative initiation mechanisms when cap-dependent translation is compromised [25,30,31,32,33].

In this study, we examined how *H. pylori* infection modulates Survivin expression at the translational level. We found that the short variant of the Survivin 5′UTR can support Survivin translation through a cap-independent mechanism, but this alternative initiation is inhibited by the oxidative stress generated by *H. pylori* GGT activity, leading to reduced Survivin protein levels. Consistently, *H. pylori* GGT suppressed cap-independent translation of a classical HIV-1 IRES sequence in comparable bicistronic reporter assays, indicating that the bacterium broadly impairs IRES-dependent mechanisms. Thus, rather than sustaining Survivin expression under stress, *H. pylori*-induced oxidative conditions compromise this alternative initiation pathway, contributing to the decline in Survivin protein levels during infection. The presence of an IRES within the short 5′UTR variant of human Survivin mRNA reveals a relevant alternative mode of translational control that could potentially be targeted to counteract *H. pylori* GGT-induced oxidative stress and restore Survivin synthesis in gastric cells.

## 2. Materials and Methods

### 2.1. Cell Lines and Culture Conditions

The human gastric cancer-derived cell line AGS (CRL-1739), the immortalized human embryonic kidney fibroblast-derived cell line HEK293T (CRL-3216), and the human cervix adenocarcinoma-derived cell line HeLa (CCL-2) were obtained from the American Type Culture Collection (ATCC, Virginia, USA). The human immortalized gastric cell line GES-1 was kindly donated by Dawit Kidane (The University of Texas at Austin, TX, USA). AGS cells were cultured in Roswell Park Memorial Institute (RPMI) 1640 medium (Gibco, Waltham, MA, USA). HEK293T, HeLa, and GES-1 cells were cultured in Dulbecco’s Modified Eagle’s Medium (DMEM) high-glucose medium (Gibco), supplemented with 2 mM glutamine, 10% fetal bovine serum (Biological Industries, Kibbutz Beit-Haemek, Israel), and antibiotics (100 U mL^−1^ penicillin, 100 µg mL^−1^ streptomycin) in a humidified atmosphere with 5% CO_2_ at 37 °C.

As previously described, *H. pylori* 26695 (ATCC 700392) and its isogenic gamma-glutamyl transpeptidase mutant (Δ*ggt*) were grown on trypticase soy agar supplemented with horse serum under microaerophilic conditions [2,34].

### 2.2. Reporter Plasmid Construction

The dl ΔEMCV and dl HIV-1 IRES bicistronic reporter plasmids were described previously [35,36,37] and kindly provided by Dr. Marcelo López (Pontificia Universidad Católica de Chile). To produce the dl Survivin 5′UTR_L_ and dl Survivin 5′UTR_S_ bicistronic plasmids, Survivin large (121 pb) and short (64 pb) 5′UTR fragments were amplified by PCR using the forward primers EcoRI-F_L_ 5′-ccgGAATTCCCCAGAAGGCCGCGGGGGGTGGAC-3′ or EcoRI-F_S_ 5′-ccgGAATTCGCGGCGCGCCATTAACCGCCA-3′, respectively, along with the reverse primer NcoI-R 5′-ccgCCAT**G**GCCGCCGCCGCCACCTCTGCC-3′ (Integrated DNA Technologies, Coralville, IA, USA), which included an extra G before the ATG initiator codon (in bold) to generate the NcoI restriction site. Following digestion with EcoRI/NcoI restriction enzymes, the amplicons were ligated together with the two larger fragments obtained by digestion of the dl HIV-1 IRES plasmid, i.e., the 5248 pb (EcoRI/XbaI) and 1656 pb (NcoI/XbaI) fragments as previously described [35].

Promoter-less plasmids dl ΔEMCV_ΔpSV40_, dl Survivin 5′UTRL_ΔpSV40,_ and dl Survivin 5′UTRS_ΔpSV40_, were obtained by digesting the corresponding bicistronic plasmid with the HindIII and MluI enzymes to eliminate the SV40 promoter region (432 bp). Cohesive ends were filled with the T4 DNA polymerase, and the resulting blunt-end plasmids were ligated under standard conditions using Promega enzymes.

Constructions were verified by sequencing analysis using an ABI PRISM 3500xL (Applied Biosystems, Waltham, MA, USA) available at the Sequencing service facility of the Pontificia Universidad Católica de Chile (Conicyt—Fondequip EQM150077).

### 2.3. Luciferase Reporter Assay

Cells were seeded at a density of 5 × 10^4^ cells/well in a 24-well plate the day before transfection. The next day, cell lines were co-transfected using the reagent Viafect^R^ (Promega, Madison, WI, USA) with the dl bicistronic plasmids (200 ng) plus a control plasmid (100 ng) constitutively expressing the β-galactosidase (LacZ) gene [38]. The β-galactosidase activity was determined in the presence of ortho-nitrophenyl-β-galactoside (ONPG), as previously described [38]. Alternatively, where indicated, HeLa cells (8 × 10^5^ cells per well in a 6-well plate) were transfected with 0.4 pmol of bicistronic (cap+, poly(A)+) or 400 ng of monocistronic (cap+/cap−, poly(A)) RNAs using the Xfect™ RNA Transfection Reagent (Takara, Kyoto, Japan), following the instructions provided by the manufacturers. After 5 h of transfection, cells were lysed, and the protein extracts were centrifuged at 11,000× *g* for 3 min. Firefly (Fluc) and Renilla (RLuc) luciferase activities in the supernatants were determined using the Dual-Glo^®^ Luciferase Assay System (Promega). Luminescence and colorimetry readouts were captured using a multiplate reader Infinite M200Pro (TECAN, Männedorf, Switzerland).

### 2.4. Western Blot Analysis

Protein extracts were prepared in a lysis buffer containing Triton 1% and 1X Complete^TM^ protease inhibitor cocktail (Roche, Mannheim, Germany), as previously described [39]. Following the manufacturer’s instructions, protein concentrations were determined using the Pierce^TM^ BCA Protein Assay reagent (Thermo Scientific, Waltham, MA, USA). Proteins (80 µg per lane) were separated by SDS–PAGE in 10% mini-gels (Bio-Rad, Hercules, CA, USA) and transferred to nitrocellulose membranes as previously described [39]. Blots were blocked with 5% milk in PBS containing 0.1% Tween-20 and then probed with different primary antibodies. Mouse monoclonal anti-Firefly luciferase was purchased from Santa Cruz Biotechnology (sc-74548, Dallas, TX, USA), rabbit anti-Renilla from Abcam (ab187338, Cambridge, UK), rabbit monoclonal anti-β-actin from Invitrogen (15G5A11/E2, Carlsbad, CA, USA), and rabbit polyclonal anti-Survivin from R&D (AF886, Minneapolis, MN, USA). The bound primary antibodies were detected with horseradish peroxidase-conjugated donkey anti-mouse (1:5000, Invitrogen SA1-100, Waltham, MA, USA) or goat anti-rabbit (1:3000, Genetex GTX213110-01) secondary antibodies. Following incubation with the EZ-ECL reagent (Biological Industries, Kibbutz Beit-Haemek, Israel) to detect HRP activity, images were captured using an ImageQuant LAS500 imager (GE Healthcare, Chicago, IL, USA).

### 2.5. Reverse Transcription and Polymerase Chain Reaction

Quantitative analysis of FLuc and RLuc mRNA levels was performed by quantitative RT-qPCR as previously described [34]. Briefly, 8 × 10^5^ cells per well were transfected with 1.5 µg of the corresponding bicistronic reporter plasmid. After 24 h, cytoplasmic fractions were obtained as previously described (29), and RNA was extracted using TriZol (Invitrogen, Carlsbad, CA, USA). Complementary DNA (cDNA) was generated using the 5X All-In-One RT Mastermix (Applied Biological Materials, #ABM.G592, Richmond, BC, Canada) and subsequently amplified with the HOT FIREPol^®^ EvaGreen^®^ qPCR mix Plus (ROX) (Solis Biodyne, Tartu, Estonia) using specific primers for FLuc (qFLuc-F 5′-CTTCGAAATGTCCGTTCGGT-3′ and qFLuc-R 5′-TAGGCTGCGAAATGCCCATA-3′), RLuc (qRLuc-F 5′-AGGTGAAGTTCGTCGTCCAACATTATC-3′ and qRLuc-R 5′-GAAACTTCTTGGCACCTTCAACAATAGC-3′), Survivin (qSurv-F 5′-CTGGCAGCCCTTTCTCAAGGA-3′ and qSurv-R 5′-GCAACCGGACGAATGCTTTT-3) and GAPDH (qGAPDH-F 5′-ATGTTCGTCATGGGTGTGAA and qGAPDH-R 5′-GGTGCTAAGCAGTTGGTGGT-3′), in a quantitative PCR thermocycler (Quant Studio^TM^ 5, Thermo Fisher Scientific, Waltham, MA, USA). Changes in mRNA levels were evaluated by the 2^−ΔΔCT^ method [40].

**Figure 1:**
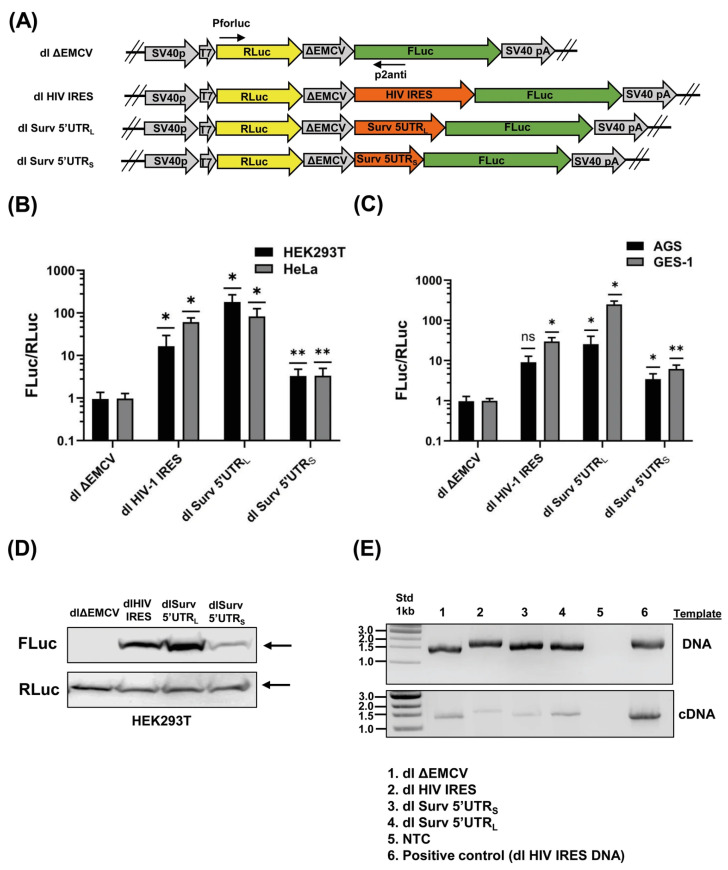
Bicistronic analysis of Survivin 5′UTR. (**A**) Schematic representation of different bicistronic dl plasmids used in this study. The control plasmid dl ΔEMCV contains a mutant version of the EMCV IRES (ΔEMCV). An SV40 promoter drives bicistronic mRNA expression of RLuc (green block arrow) and Fluc (yellow block arrow) genes. The SV40 polyadenylation signal is present at 3′. HIV-1 IRES and Survivin 5′UTR variants (orange block arrows) were cloned between ΔEMCV and FLuc sequences. Also, the relative recognition sites for Pforluc and p2anti primers are indicated. (**B**,**C**). The cell lines AGS, GES-1, HEK293T, and HeLa were co-transfected with the reporter plasmids dl ΔEMCV, dl HIV-1 IRES, dl Surv 5′UTR_L,_ or dl Surv 5′UTR_S_ combined with the control plasmid pON (beta-galactosidase). After 24 h, protein extracts were prepared to determine luciferase reporter activities. Bars represent values of normalized luminescence (RLuc/FLuc), compared with the control condition (dl ΔEMCV) (means ± SD, n = 3, * *p* ≤ 0.05, ** *p* ≤ 0.01). (**D**) HEK293T cells were transfected with the indicated bicistronic dl plasmids (ΔEMCV, HIV-1 IRES, Surv 5′UTR_L,_ and Surv 5′UTR_S_). Following 24 h of transfection, total protein extracts were prepared and separated by SDS-PAGE on 10% gels. RLuc and FLuc protein expression was evaluated by Figure S1). A representative blot is shown. (**E**) HeLa cells were transfected with the dl bicistronic plasmids (lanes 1–4). After 24 h of transfection, plasmid DNA or bicistronic mRNAs were recovered and amplified by PCR (**upper** panel) or RT-PCR (**lower** panel), respectively, using the primers PforLuc and p2anti. Amplicons were resolved by agarose gel electrophoresis (1%). Samples without DNA or cDNA addition (No template control NTC, lane 5) were amplified as a negative control. As a positive control, samples with 100 ng of dl HIV-1 IRES were amplified (lane 6). A 1Kb DNA ladder was also included (Figure S2)).

To analyze the integrity of the bicistronic plasmids once they are inside cells, DNA was recovered from transfected HeLa cells (24 h) with the E.Z.N.A.^®^ Tissue DNA kit (Omega Bio-Tek, Norcross, GA, USA). Also, cDNA was prepared as mentioned above 24 h post-transfection. Then, bicistronic DNA and cDNA were amplified in an end-point thermocycler (Veriti^TM^, Thermo Fisher Scientific) under standard conditions using the primers F-RLuc-IRES (Pforluc) 5′-CATGACTTCGAAAGTTTATGATC-3′ and R-FLuc-IRES (p2anti) 5′-TCTCTTCATAGCCTTATGCAGTTG-3′, as previously described [41] (see Figure 1A). Amplicons were analyzed by electrophoresis in 1% agarose gels and compared with a 1 kb molecular weight standard (New England Biolabs, Ipswich, MA, USA).

### 2.6. In Vitro Transcription

For in vitro transcription of bicistronic DNAs, T7-DNA templates were obtained by digesting the dl ΔEMCV, dl Surv5UTR_S/L_, or dl HIV-1 IRES plasmids with the restriction enzyme BamHI and then purified in an RNAse-free environment, as previously described [36]. Then, capped and polyadenylated RNAs (cap+, poly(A)+) were synthesized using the mMESSAGE mMACHINE T7 Transcription Kit (Invitrogen, Carlsbad, CA, USA) according to the manufacturer’s instructions (250 ng T7-DNA template, 90 min, 37 °C). After transcription, the template DNA was eliminated by digesting with the Turbo DNase provided by the manufacturer. Then, RNAs were immediately poly(A) tailed with the *E. coli* poly(A) polymerase (E-PAP, New England BioLabs, Ipswich, MA, USA) for 30 min at 37 °C, precipitated with 7.5 M LiCl, and finally resuspended at a concentration of 50 ng/µL in nuclease-free water.

For monocistronic RNA synthesis, T7-DNA amplicons were obtained by PCR using the dl Surv5′UTR_S_ plasmid as a template using the forward primer T7-SurvS-F 5′-AGTACTTAATACGACTCACTATAGCGGCGCGCCATTAACCGCCAGATTTG-3′along with the reverse primer T7-SVPolA-R 5′-TACCACATTTGTAGAGGTTTTACTTGCTTT-3′. A-capped RNAs were obtained by replacing the 2× NTP/CAP buffer of mMESSAGE mMACHINE™ T7 Transcription Kit with a similar mixture including 15 mM ATP, 15 mM CTP, 15 mM UTP, 3 mM GTP, and 12 mM of the non-functional cap analog AP_3_G (Jena Bioscience, Jena, Germany).

### 2.7. In Vitro Translation

In vitro uncoupled translations were performed using the nuclease-treated rabbit reticulocyte lysate (RRL) system (Promega), as previously described [42]. Briefly, standard reactions in a final volume of 25 µL, adjusted with endonuclease-free water, included 35% (*v*/*v*) RRL, 20 µM amino acids, 20 U of the RNase inhibitor RNasin^®^ (Promega), and 100 ng of bicistronic RNAs (cap+, poly(A)+). Where indicated, the final concentrations of potassium acetate (KOAc) and magnesium acetate (MgOAc_2_) were adjusted to concentrations ranging from 0–200 mM or 0–2 mM, respectively. The translation reaction was incubated at 30 °C for 90 min. Finally, firefly and Renilla luciferase activities were measured using the Dual-Glo Luciferase Assay System (Promega, Madison, WI, USA). Alternatively, coupled transcription/translation using the bicistronic DNA BamHI-digested with the enzyme was performed using the TnT^®^ Quick Coupled Transcription/Translation System (Promega, Madison, WI, USA), following the manufacturer’s instructions.

### 2.8. siRNA Transfection

HeLa cells were seeded at a density of 4 × 10^5^ cells/well in a 6-well plate on the day before transfection. The next day, 0.4 pmol (400 ng approximately) of bicistronic RNA (cap+, poly(A)+), obtained from in vitro transcription of dl HIV-1 IRES or dl Survivin 5′UTR_S_ BamHI-digested plasmids, were co-transfected with 0,10, 20, or 40 nM of either a negative control (Ambion^®^ Silencer Negative Control #1 siRNA, Thermo Fisher Scientific) or an effective siRNA targeting the RLuc open reading frame (MISSION^®^ esiRNA EHURLUC, Sigma-Aldrich, St. Louis, MO, USA), using the Xfect™ RNA Transfection Reagent (Takara), following the instructions provided by the manufacturer. RLuc and FLuc luminescence activities were determined at 5 h post-transfection as described above. Alternatively, HeLa cells were co-transfected with 0.4 µg of the dl HIV-1 IRES or dl Surv 5′UTR_S_ plasmids combined with the siCtl or siRLuc at the indicated concentrations. After 24 h, cell lysates were obtained, and luciferase activity was determined.

### 2.9. Infection of AGS Cells

For the reporter assay, AGS cells were transfected with bicistronic plasmids and the pON control plasmid under conditions like those described above. After 24 h of transfection, the gastric cells were infected with *H. pylori* 26695 wild-type or the Δ*ggt* isogenic mutant at a multiplicity of infection (MOI) of 1:50 or 1:100 for 24 h, as previously described in our studies [2]. Subsequently, cell lysates were collected, and the activities of luciferase and β-galactosidase were measured. In parallel, cells were treated with arsenic trioxide (ATO) at concentrations ranging from 1 to 10 µM.

### 2.10. Statistical Analysis of Data

All data are expressed as the means ± standard deviation of at least three independent experiments. Data were processed using GraphPad Prism Software v10.6.1 (892), (San Diego, CA, USA, www.graphpad.com). The statistical significance of differences was determined using the Wilcoxon–Mann–Whitney test for non-parametric data and was considered significant at *p* < 0.05.

## 3. Results

### 3.1. The Survivin 5′UTRs Drive Translation in a Bicistronic Assay

The possibility that Survivin 5′ untranslated region (5′UTR) recruits the ribosome in a cap-independent manner has not been addressed to date. To evaluate this possibility, we performed a classical bicistronic reporter assay using two variants of Survivin 5′UTR, which were cloned into the bicistronic plasmid dl ΔEMVC employed in previous related studies [41,43]. The large variant (L, 121 pb) represents a conserved extension found in the Survivin 5′UTRs of several vertebrates, including humans, and the short fragment (S, 64 pb), which is present as the most prevalent variant form in humans [34]. As shown (Figure 1A), the sequence of the dl ΔEMVC plasmid permits the expression of a bicistronic mRNA that encodes for Renilla and Firefly luciferases (RLuc and FLuc, respectively). Additionally, this plasmid contains regulatory elements, including the SV40 virus promoter and a polyadenylation signal, which are essential for the transcription and production of mature bicistronic mRNAs in transfected cell lines. Of note, the first cistron RLuc is translated in a cap-dependent manner; however, the translation of the second cistron, FLuc, is greatly impeded due to the presence of a highly structured defective encephalomyocarditis virus (EMCV) internal ribosome entry site (IRES) sequence (ΔEMCV), which blocks ribosome reinitiation and readthrough [41] (see Figure 1A). Thus, translation of the second cistron FLuc is possible only if the sequence cloned between the ΔEMVC and FLuc regions behaves like an IRES element, recruiting the ribosome in a cap-independent manner. As shown in Figure 1A, we transfected four human cell lines with the above-mentioned constructs. Also, we included a positive control plasmid, which contains the HIV-1 IRES sequence downstream of the ΔEMCV region (dl HIV-1 IRES plasmid) [43]. Reporter assays in these different cell backgrounds (Figure 1B,C) revealed a significant increase in the FLuc/RLuc luminescence ratio when cells were transfected with either the dl HIV-1 IRES, dl Surv 5′UTR_L_, or dl Surv 5′UTR_S_ plasmids compared with the dl ΔEMVC control plasmid (FLuc/RLuc ratio = 1). However, reporter activity was notably higher when the cells were transfected with the dl Survivin 5′UTR_L_ plasmid. Thus, these results indicate that Survivin 5′ UTRs can be translated in a cap-independent manner.

Furthermore, we evaluated the expression of FLuc and RLuc proteins in HEK293T cells transfected with the dl plasmids by Western blotting. As shown (Figure 1D), immunodetection of FLuc in total protein extracts was more evident when cells were transfected with either the dl Surv 5′UTR_L_ or control dl HIV-1 IRES plasmids. This result was consistent with that observed in the reporter assays, where the cap-independent activity of the short Survivin 5′UTR_S_ was modest compared to the large variant.

Next, to rule out that the observed cap-independent activity of the Survivin 5′UTR_L_ was due to a possible rearrangement of the plasmid inside the cells or that the bicistronic mRNA produced had undergone alternative splicing, we isolated total DNA and RNA from HeLa cells after 24 h of transfection with each dl plasmid. As shown in Figure 1E, PCR products obtained using the Pforluc and p2anti primers (positioned with arrows in Figure 1A) were analyzed by electrophoresis in agarose gels. However, no amplicons or products of sizes other than those expected were detected, whether the recovered bicistronic plasmid DNAs (upper panel) or bicistronic cDNA (lower panel) were used as templates. This result can be taken as evidence that the observed cap-independent expression of Fluc was not due to the rearrangement of the plasmids or the mRNAs produced.

### 3.2. The Survivin 5′UTR_L_ Contains a Region with Cryptic Promoter Activity

**Figure 2:**
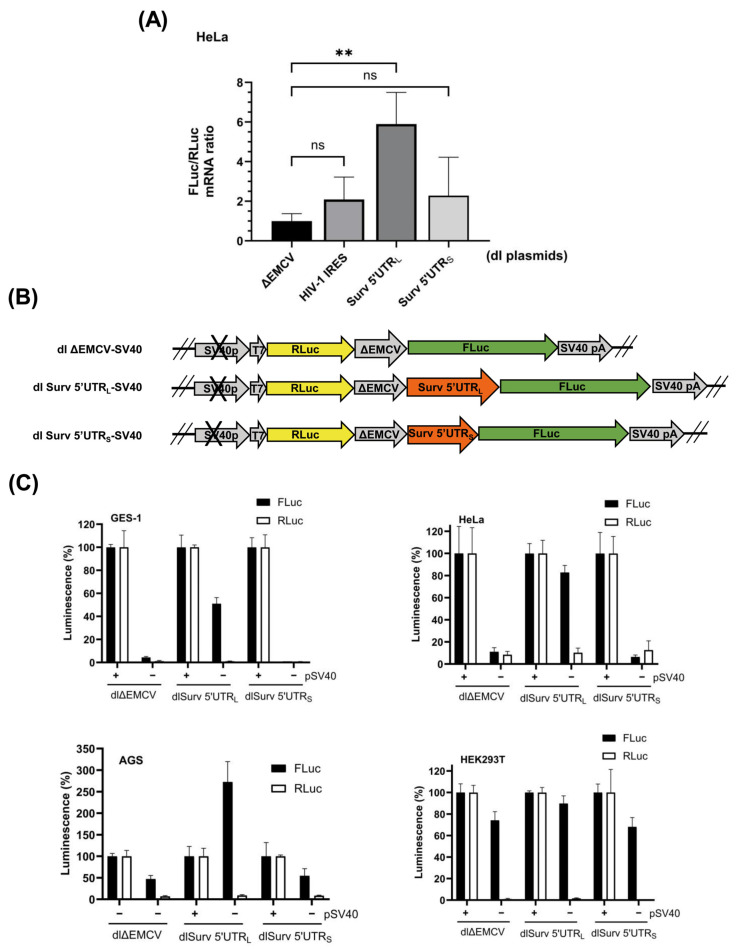
The Survivin 5′UTR_L_ presented cryptic promoter activity. (**A**) HeLa cells were transfected with the indicated dl bicistronic plasmids (dl ΔEMCV, dl HIV-1 IRES, dl Surv 5′UTR_L,_ and dl Surv 5′UTR_S_). After 24 h of transfection, mRNA levels of FLuc were evaluated by RT-qPCR and normalized to RLuc mRNA levels. Data were expressed relative to values obtained for the transfection with the dl ΔEMCV plasmid (means ± SD, n = 3; ** *p* ≤ 0.01, ns: non-significant). (**B**) Schematic representation of promoter-less bicistronic plasmids (ΔpSV40) used in this study. (**C**) The cell lines GES-1, HeLa, AGS, and HEK293T were co-transfected with the reporter plasmids (dl ΔEMCV, dl Surv 5′UTR_L_ and dl Surv 5′UTR_S_) or their respective promoter-less plasmids (dl ΔEMCV_ΔpSV40_, dl Survivin 5′UTR_L-ΔpSV40_ and dl Survivin 5′UTR_S-ΔpSV40_) combined with the control plasmid pON (beta-galactosidase). After 24 h, protein extracts were prepared to determine luciferase reporter activities. Bars represent values of normalized luminescence (FLuc or RLuc) of promoter-less plasmids, compared with the parental plasmid (100% luminescence) (means ± SD, n = 3).

Since the Surv 5′UTR_L_ displayed FLuc activity in the reporter assays, which was even higher than observed for the HIV-1 IRES, we first wondered if this augmented expression was a consequence of cryptic promoter activity. Thus, we evaluated the mRNA levels of FLuc and RLuc cistrons by quantitative reverse transcription PCR (RT-qPCR). As shown (Figure 2A), the relative expression of FLuc normalized to RLuc mRNA in HeLa cells transfected with the dl Surv 5′UTR_L_ plasmid was 6-fold higher than that observed with the control plasmid dl ΔEMCV. These results suggest that the Survivin 5′UTR_L_ DNA sequence behaves like a cryptic promoter, generating a monocistronic FLuc RNA. Next, to confirm or discard this possibility, we performed reporter assays using the SV40 promoter-less versions of the dl ΔEMVC, dl Surv 5′UTR_L_, and dl Surv 5′UTR_S_ plasmids (see Figure 2B). As shown in reporter assays (Figure 2C), an almost total loss of RLuc and FLuc activities was observed in GES-1, HeLa, and AGS cells when these were transfected with the promoter-less versions of the dl ΔEMVC and dl Survivin 5′UTR plasmids. However, the dl Surv 5′UTR_L_ promoter-less plasmid retained a considerable percentage of FLuc activity in these cell lines (51.1 ± 5.2%, 82.8 ± 6.3%, and 239 ± 50%, respectively), even though RLuc activity was abolished. Interestingly, reporter assays in HEK293T cells yielded an unexpected result. In this cell line, all promoter-less plasmids displayed elevated FLuc activity, although RLuc activity was suppressed (Figure 2C). This observation could be interpreted as indicating that the defective EMCV IRES sequence behaves as a cryptic promoter in these cells, which were immortalized using a sheared adenovirus 5 DNA [44]. These results confirm that the Survivin 5′UTR_L_ sequence retains considerable promoter activity in various cell contexts.

### 3.3. Survivin 5′UTR_S_ Exhibits Cap-Independent Activity

**Figure 3:**
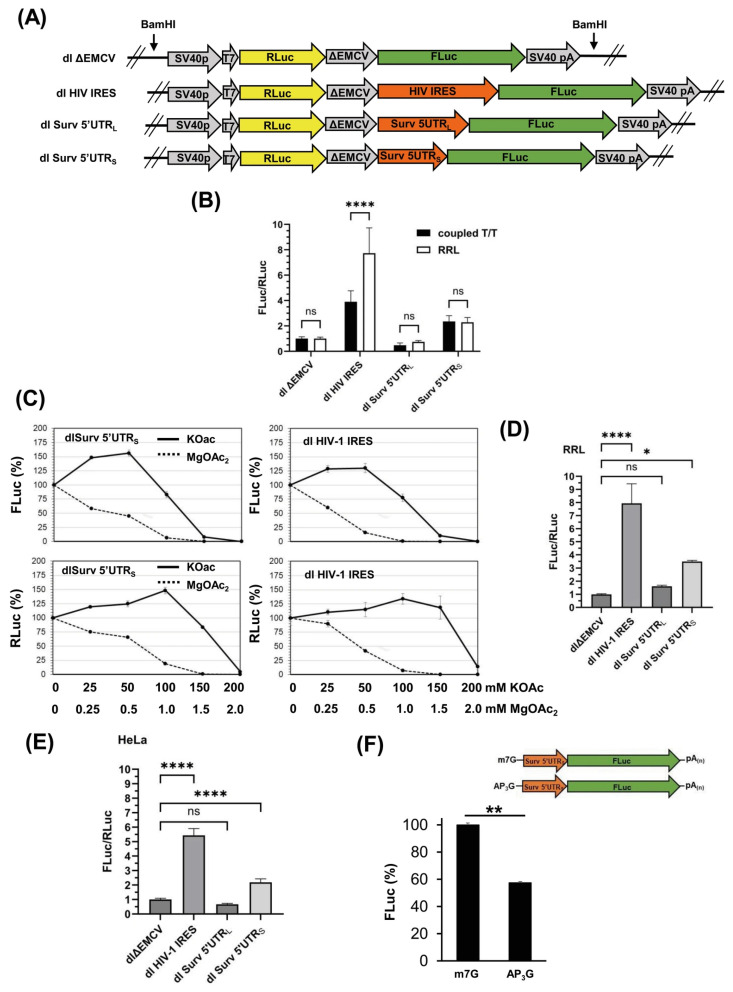
In vitro T7 transcription and translation of dl bicistronic plasmids. (**A**) Schematic representation of the dl bicistronic plasmids digested with the BamHI restriction enzyme, suitable for in vitro T7 RNA polymerase transcription. (**B**) Coupled and uncoupled transcription/translation reactions (black and white bars, respectively) were performed using the indicated bicistronic DNAs as templates. Bars represent values of the luminescent ratios RLuc/FLuc, compared with the control condition (dl ΔEMCV) (means ± SD, n > 3, **** *p* < 0.0001, ns: non-significant). (**C**) Uncoupled transcription/translation reactions were performed by RRL using the dl Surv 5′UTR_S_ or dl HIV-1 IRES BamHI-digested plasmids as indicated, including the addition of different concentrations of KOAc (0, 25, 50, 100, 150, and 200 mM) or Mg(OAc)_2_ (0, 0.25, 0.5, 1.0, 1,5 and 2.0 mM). Curves represent the percentage of luciferase activity (Fluc or RLuc) compared with the condition without additional external salts (means ± SD, n = 3). (**D**) As indicated, uncoupled transcription/translation reactions were performed by RRL using the BamHI-digested bicistronic plasmids, including an optimized KOAc concentration (50 mM). Bars represent values of the luminescence ratios RLuc/FLuc, compared with the control condition (ΔEMCV) (means ± SD, n > 3, * *p* ≤ 0.05, **** *p* ≤ 0.0001, ns: non-significant). (**E**) HeLa cells were transfected with 0.5 µg of bicistronic RNAs (cap+, poly(A)) as indicated. After 5 h, protein extracts were prepared to determine luciferase reporter activities. Bars represent values of luminescence ratios FLuc/RLuc, compared with the control condition (dl ΔEMCV, FLuc/RLuc = 1) (means ± SD, n > 3, **** *p* < 0.0001, non-significant). (**F**) HeLa cells were transfected with 0.5 µg of capped (m7G) or uncapped (AP_3_G) monocistronic Surv 5′UTR_S_-FLuc RNA. After 5 h, protein extracts were prepared to determine Firefly luciferase activity. Bars represent the percentage of FLuc luminescence (means ± SD, n = 3, ** *p* < 0.01).

Since the expression of the bicistronic mRNAs from transfected plasmids revealed the presence of cryptic promoter activity in the Survivin 5′UTR_L_, the following approach was to determine the presence of cap-independent activity of Survivin 5′UTR variants by translating the bicistronic RNAs using the rabbit reticulocyte lysates (RRL). With this in mind, coupled and uncoupled transcription/translation assays were performed using the dl plasmids linearized with the BamHI restriction enzyme or the bicistronic RNAs obtained in vitro, respectively. Of note, dl plasmids harbor a T7 promoter upstream of the RLuc gene, which allows in vitro transcription by the T7 polymerase (Figure 3A). As shown (Figure 3B), coupled or uncoupled transcription/translation resulted in an elevated FLuc/RLuc ratio when the dl HIV-1 IRES plasmid was used as template (3.9- and 7.7-fold, respectively), although changes for the Survivin 5′UTR_S_ were modest (2.4- and 2.8-fold, respectively). Conversely, Survivin 5′UTR_L_ showed FLuc/RLuc ratios slightly lower than the control plasmid (0.47- and 0.74-fold, respectively). Next, we investigated whether this cap-independent translation was influenced by changes in K^+^ or Mg^+2^ concentrations, as has been described for other IRES sequences [42]. As shown (Figure 3C), FLuc activity responded positively to concentrations of KOAc between 25–50 mM; however, MgOAc_2_ was inhibitory at all concentrations tested when bicistronic mRNAs (from T7 RNA polymerase-mediated transcription of dl HIV-1 IRES and dl Surv5′UTR_s_ BamHI-digested plasmids) were translated in uncoupled translation reactions using RRL. Of note, the selective effect of KOAc was noticeable at a concentration of 150 mM when cap-independent translation of Fluc driven by HIV-1 IRES or Surv5′UTR_s_ sequences was inhibited by more than 95%; however, cap-dependent translation of RLuc remained close to 100% compared to the control condition (Figure 3C).

Additionally, we performed a reporter assay to determine the extent of translation of the bicistronic mRNAs under a standardized salt concentration of 50 mM KOAc without additional MgOAc_2_ (Figure 3D), which increased the FLuc/RLuc ratio for dl HIV-1 IRES and dl Survivin 5′UTR_S_ to 7.9- and 3.5-fold, respectively. Also, we transfected HeLa cells with bicistronic RNAs (cap+, poly(A)+) and analyzed the FLuc and RLuc activities after 5 h. As shown in Figure 3E, the results of RNA transfection were consistent with translation in RRL; although FLuc/RLuc ratios were modest for the HIV-1 IRES and Survivin 5′UTR_S_ cap-independent activity (5.4-fold and 2.2-fold, respectively). Finally, we wondered to what extent the translation of the monocistronic version of FLuc driven by the Survivin 5′UTR_S_ could be supported by the 5′cap modification. Thus, we transfected HeLa cells with de monocistronic Surv 5′UTR_S_-FLuc RNA capped (m7G) or uncapped (AP_3_G). As shown in Figure 3F, the FLuc activity of the uncapped RNA reaches 58% compared to capped RNA. These results confirm that the uncapped Survivin 5′UTR short region can sustain translation in this cellular context.

### 3.4. siRNA-Mediated Destabilization of Bicistronic mRNAs

**Figure 4:**
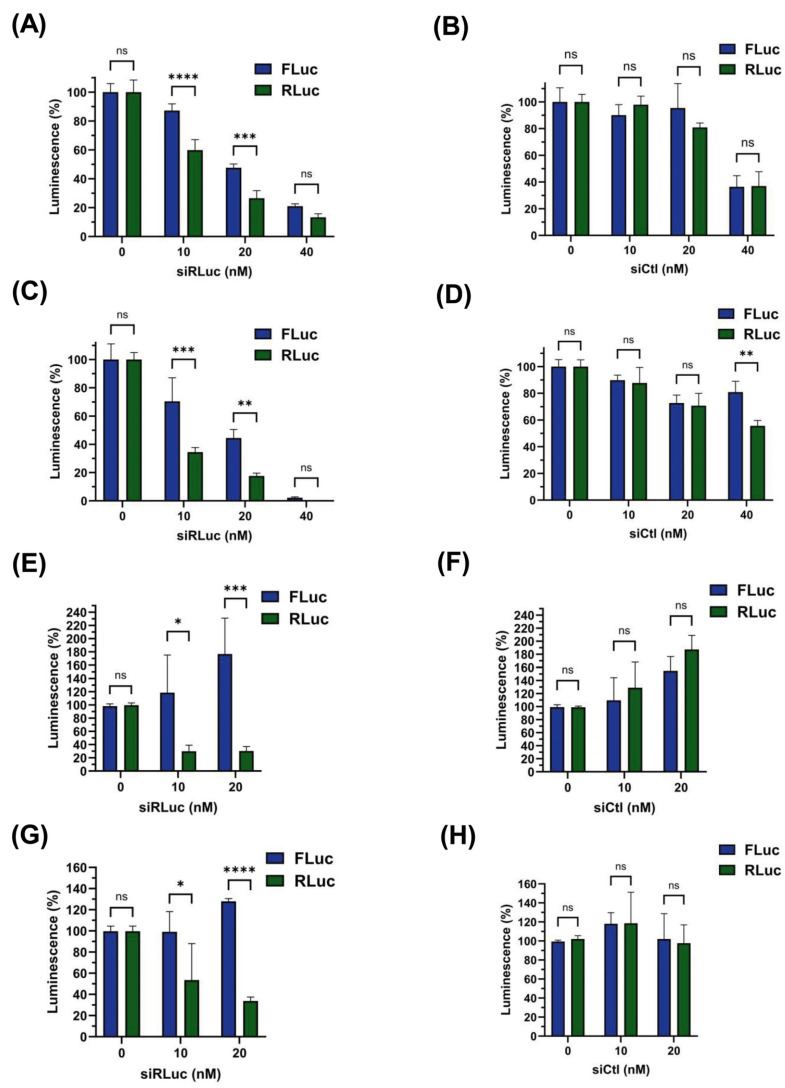
Silencing of Renilla luciferase expression by a short interfering RNA. HeLa cells were transfected with the indicated bicistronic RNAs ((**A**,**B**) dl HIV-1 IRES RNA or (**C**,**D**) dl Surv 5′UTR_S_ RNA) or the dl bicistronic plasmids ((**E**,**F**) dl HIV-1 IRES DNA or (**G**,**H**) dl Surv 5′UTR_S_ DNA) in combination with different concentrations of an effective siRNA against the RLuc coding region (**A**,**C**,**E**,**G**) or a scrambled control siRNA (**B**,**D**,**F**,**H**) at various concentrations (0, 10, 20 or 40 nM). Cell lysates were prepared to determine luciferase reporter activities after 5 h (for RNA transfections) or 24 h (for DNA transfections). Bars represent the percentage of luciferase activity FLuc (blue bars) and RLuc (green bars), compared with the control condition (means ± SD, n = 3, * *p* < 0.05, ** *p* < 0.01, *** *p* < 0.001, **** *p* < 0.0001, ns: non-significant).

Next, we developed another assay that used a short interfering RNA directed against the first cistron RLuc (siRLuc) to destabilize the 5 ‘region of the bicistronic mRNA and thus silence cap-dependent translation in HeLa cells. This methodology has been widely used to confirm IRES activity in other sequences [41,45]. As can be seen in Figure 4, the co-transfection of bicistronic mRNAs (cap+, poly(A)+) carrying either the HIV-1 IRES (Figure 4A) or Survivin 5′UTR_S_ (Figure 4C) showed a significant decrease in RLuc activity in response to increasing concentrations of the siRLuc; however, the FLuc activity was less susceptible to the action of siRLuc, although a considerable reduction was detected upon exposure to higher concentrations of siRLuc (20–40 nM). As expected, the control siRNA (siCtl) did not generate a significant drop in the luminescence of RLuc or FLuc at 10 nM, but did reduce activity at higher concentrations (20–40 nM) (Figure 4B,D).

Since co-transfection of the siRNA and the bicistronic RNAs appeared to strongly compromise both cistrons, possibly due to enhanced RNA destabilization, we conducted a similar experiment by transfecting HeLa cells with the bicistronic plasmids dl HIV-1 IRES and dl Survivin 5′UTR_S_, using these constructs as controls to maintain mRNA levels over time. As shown, introducing siRLuc into HeLa cells transfected with bicistronic plasmids significantly reduced the RLuc activity in both cases; however, the FLuc activity was not affected by any of the siRLuc concentrations used (Figure 4E and Figure 4G, respectively). These results further confirm the presence of a cap-independent regulatory sequence in the Survivin 5′UTR_S_. As expected, the control siRNA (siCtl) did not generate a significant drop in the luminescence of RLuc or FLuc at any of concentrations tested (Figure 4F,H).

### 3.5. The Cap-Independent Translation Is Sensitive to Oxidative Stress Produced by H. pylori Gamma-Glutamyl Transpeptidase Activity and a Prooxidant Agent

**Figure 5:**
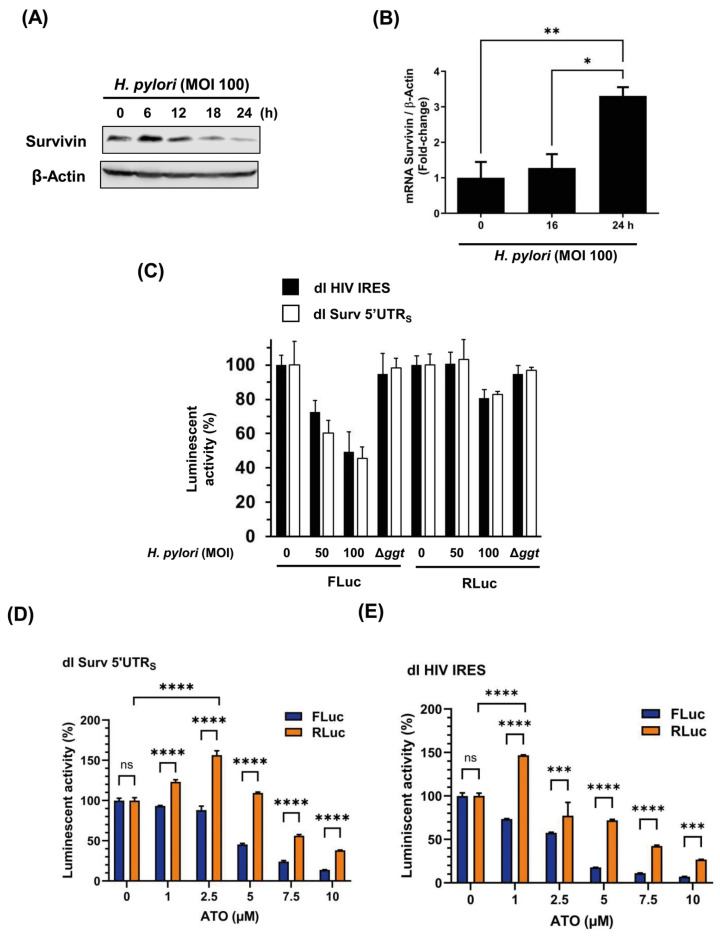
Effect of *H. pylori* infection on cellular and viral cap-independent translation in AGS cells. (**A**) Cells were infected with *H. pylori* 26695 (MOI 100, 24 h) and cell protein extracts were subjected to Western blot analysis (Figure S3), using antibodies against human Survivin and β-actin. A representative blot is shown. (**B**) Evaluation of changes in Survivin mRNA levels in cells infected with *H. pylori* 26695 (MOI100, 24 h) by RT-qPCR (means ± SD, n = 3, * *p* < 0.05, ** *p *< 0.01). (**C**) AGS cells were transfected with dl Surv 5′UTR_S_ or dl HIV-1 IRES plasmids for 24 h. Then, cells were infected with *H. pylori* 26695 (MOI 50–100) or its isogenic mutant Δ*ggt* (MOI 100). Following 24 h of infection, protein extracts were prepared to determine FLuc and RLuc luminescence activities. Bars represent the percentage of luciferase activity compared to the control condition without bacterial infection (means ± SD, n = 3). (**D**,**E**). AGS cells were transfected with dl Surv 5′UTR_S_ (**D**) or dl HIV-1 IRES plasmids (**E**) for 24 h. Then, cells were treated with different concentrations of ATO (1, 2.5, 5, 7.5, or 10 µM) for 24 h. Protein extracts were prepared to determine the luminescent activities of FLuc and RLuc. Bars represent the percentage of luciferase activity compared with the control condition without treatment (means ± SD, n = 3, *** *p* ≤ 0.001, **** *p* ≤ 0.0001, ns: non-significant).

As mentioned, Survivin is a protein expressed in the normal gastric epithelium [2,3]. Previous results have shown that Survivin protein levels are decreased in human gastric epithelium and gastric cells in vitro due to *H. pylori* infection (Figure 5A), primarily due to oxidative stress induced by the bacterial gamma-glutamyl transpeptidase (GGT) activity [2]. However, the mechanism by which GGT activity reduces Survivin protein levels was not fully elucidated, since a transcriptional inhibition of BIRC5 is not observed in gastric cells infected in vitro (Figure 5B). For this reason, we wondered whether *H. pylori* infection could modulate the cap-independent translation of FLuc driven by the Survivin 5′UTR_S_ in our experimental model. With this in mind, AGS gastric cells were transfected with the dl bicistronic vectors (dl HIV-1 IRES and dl Surv5′UTR_S_) and subsequently infected with *H. pylori* at different multiplicities of infection (MOI). As shown in Figure 5C, this was indeed the case. FLuc activity driven by the Survivin 5′UTR_S_ and HIV-1 IRES sequences was diminished in an MOI-dependent manner; however, RLuc activity was resistant to *H. pylori* infection. Conversely, FLuc activity driven by both the Survivin 5′UTR or HIV-1 IRES sequences was unaffected when AGS cells were infected with the Δ*gg*t mutant. Furthermore, we incorporated arsenic trioxide (ATO), a well-known pro-oxidant molecule [39], into this reporter assay. As expected, oxidative stress mediated by ATO treatment significantly reduced cap-independent activity (FLuc) without a noticeable decrease in RLuc activity (Figure 5D). This effect was most pronounced at concentrations ranging from 1 µM to 5 µM of ATO. However, at a higher concentration (7.5–10 µM), RLuc activity was also reduced in response to ATO-induced cytotoxicity, although to a lesser extent. Notably, low doses of ATO stimulated cap-dependent translation of RLuc. Taken together, these results demonstrate the inhibitory effect of oxidative stress on cap-independent translation.

## 4. Discussion

Survivin is a multifunctional inhibitor-of-apoptosis protein whose expression is tightly regulated under physiological conditions but frequently deregulated in cancer. While largely absent from most differentiated adult tissues, Survivin is maintained in epithelial compartments with high cellular turnover, such as the gastric mucosa, where it contributes to epithelial integrity, controlled proliferation, and resistance to apoptosis [46]. Chronic *Helicobacter pylori* infection is associated with a progressive loss of Survivin expression in gastric epithelial cells, correlating with increased apoptosis, impaired tissue regeneration, and mucosal damage [2,3,47]. Previous studies linked this phenomenon to oxidative stress generated by the bacterial gamma-glutamyl transpeptidase (GGT); however, the post-transcriptional mechanisms underlying Survivin downregulation remained poorly defined.

In this study, we identify a short variant of the human Survivin 5′ untranslated region (5′UTR_S_) that supports cap-independent translation. Using complementary and orthogonal approaches—including DNA- and RNA-based bicistronic reporter assays, promoter-less constructs, in vitro translation systems, uncapped RNA transfection, and siRNA-mediated destabilization of the first cistron—we demonstrate that this short 5′UTR harbors a functional cap-independent translational regulatory element. Importantly, this activity is selectively inhibited by oxidative stress induced either by *H. pylori* GGT activity or by treatment with the pro-oxidant arsenic trioxide (ATO), whereas cap-dependent translation remains relatively preserved under moderate stress conditions.

A critical aspect of our analysis was the distinction between the short and long Survivin 5′UTR variants. The longer variant (5′UTR_L_), which predominates in several vertebrate species, exhibited substantial cryptic promoter activity in multiple cell lines. This transcriptional activity confounds the interpretation of DNA-based bicistronic assays and likely explains inconsistencies in earlier studies that did not discriminate between transcriptional and translational effects. When transcriptional artifacts were excluded using promoter-less constructs and RNA-based assays, the long variant did not support cap-independent translation. In contrast, cap-independent activity was consistently observed for the short 5′UTRs, which is also the most prevalent Survivin 5′UTR variant in human cells [34]. These findings indicate that the short 5′UTR variant primarily mediates translational regulation of Survivin via cap-independent mechanisms in humans.

Although viral internal ribosome entry sites (IRES) are typically several hundred nucleotides long and structurally complex, increasing evidence indicates that cellular cap-independent translation elements can be considerably shorter and function in a context-dependent manner [48,49,50]. Indeed, many cellular mRNAs encoding regulators of cell survival, proliferation, and stress responses rely on compact IRES-like elements or cap-independent regulatory regions with modest translational efficiency [48,49,50]. Accordingly, the Survivin 5′UTR_S_ should be viewed not as a canonical viral-type IRES, but rather as a functional cap-independent translation element whose activity depends on cellular context and regulatory factors. This distinction is essential, as cellular IRES-like elements often display lower activity than viral counterparts and are highly sensitive to changes in the intracellular environment.

Cap-independent translation is frequently proposed as a mechanism that sustains the expression of specific mRNAs when global cap-dependent translation is compromised, such as during stress, apoptosis, or metabolic imbalance. In cancer cells, where oxidative stress, hypoxia, and nutrient limitation are common, this mechanism may contribute to sustained Survivin expression, thereby promoting resistance to apoptosis and tumor progression. In this context, Survivin joins a growing group of cellular mRNAs encoding anti-apoptotic or pro-survival proteins—including XIAP, Cyclin D1, c-Myc, and p53—that are subject to translational regulation through cap-independent mechanisms [50]. Thus, the presence of a cap-independent regulatory element within the Survivin 5′UTR provides a plausible explanation for how Survivin expression may be maintained under adverse conditions in transformed cells.

A central finding of this study is that oxidative stress inhibits, rather than enhances, cap-independent translation driven by the Survivin 5′UTR_S_ in gastric epithelial cells. This inhibitory effect was also observed for a canonical HIV-1 IRES, indicating that *H. pylori* GGT activity broadly interferes with IRES-dependent translation rather than selectively targeting Survivin. While previous reports have shown that oxidative stress can stimulate IRES activity in specific cellular contexts [51], these effects appear to be highly cell-type dependent and influenced by the nature, intensity, and duration of the stress [52,53]. Our results indicate that, in gastric epithelial cells, oxidative stress creates a translational environment that is unfavorable for cap-independent initiation.

**Figure 6:**
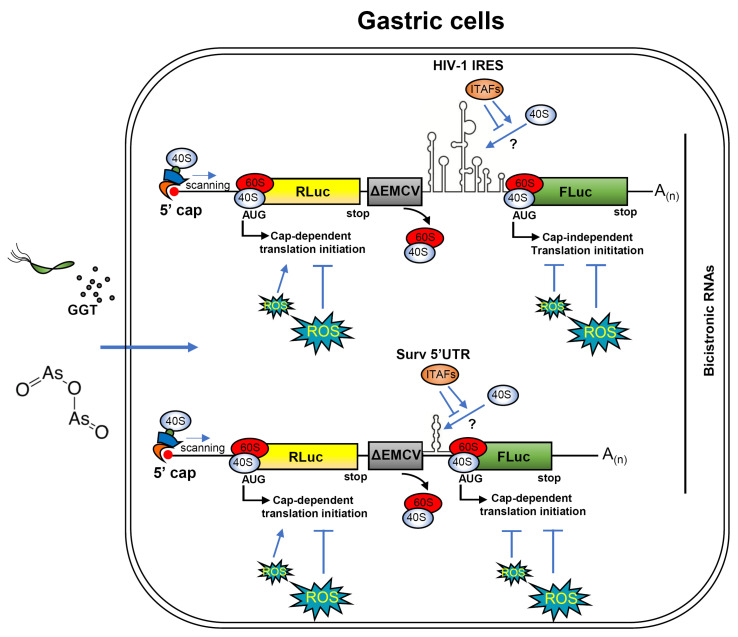
Schematic representation of cap-dependent translation inhibition by ROS in gastric cells. Bicistronic RNAs containing either the HIV-1 IRES or the Survivin 5′UTR sequences are translated in both cap-dependent and cap-independent manners. Cap-dependent translation of the first cistron, RLuc, occurs when the 40S subunit is recruited at the 5′ cap with the assistance of elongation factors. The complex then scans the 5′UTR until it reaches the first initiation codon, AUG, where the catalytically active ribosome is assembled. Once the RLuc ORF is translated, the defective ΔEMCV IRES causes the ribosome to drop off the RNA sequence. Thus, FLuc translation is only possible if a cap-independent sequence, such as HIV-1 IRES or Survivin 5′UTR, is present upstream of the cistron, enabling the recruitment of the 40S subunit. In AGS gastric cells, *H. pylori* GGT activity and ATO treatment induce ROS production, which affects translation efficiency. At low doses of ATO, cap-dependent translation is stimulated; however, cap-independent translation is sensitive to ROS at all tested doses. A possible mechanism for this observation could involve ITAFs, which are responsible for recruiting the 40S subunit to secondary IRES structures. The secondary structure of the Survivin 5′UTR was predicted using the RNAstructure v6.5 online tool (Mathews lab), while the secondary structure of the HIV-1 IRES was adapted from Ohlmann et al. [66].

Although we did not directly identify the molecular mediators involved, several IRES trans-acting factors (ITAFs), including HuR, hnRNPA1, PTB, PSF, and PDCD4, have been reported to repress IRES-dependent translation in response to oxidative or endoplasmic reticulum (ER) stress [50,54,55,56,57]. Notably, *H. pylori* infection and GGT activity have been linked to activation of the PERK–eIF2α signaling pathway [58], and arsenic trioxide is a well-established inducer of ER stress and eIF2α phosphorylation [59,60]. Emerging evidence suggests that eIF2α phosphorylation does not simply suppress global translation but can selectively inhibit certain viral and cellular IRES elements [61,62]. Together, these observations support a model in which redox-dependent stress signaling interferes with cap-independent translation initiation through modulation of ITAF availability or function (Figure 6).

From a pathophysiological perspective, inhibition of cap-independent translation may have profound consequences for gastric epithelial homeostasis. Chronic *H. pylori* infection is characterized by increased epithelial apoptosis, impaired regeneration, and progression toward atrophic gastritis and intestinal metaplasia [47,63,64,65]. Survivin is a key anti-apoptotic factor required to maintain epithelial integrity in tissues with high cellular turnover [46], and its depletion has been directly linked to increased apoptosis in gastric epithelial cells during infection [2,3,47]. Therefore, suppression of cap-independent translation of Survivin—and potentially other survival-associated proteins—may exacerbate the imbalance between cell loss and renewal, contributing to epithelial damage and disease progression.

In summary, our study identifies a short cap-independent translational regulatory element within the human Survivin 5′UTR and demonstrates that this mechanism is selectively inhibited by oxidative stress generated by *H. pylori* γ-glutamyl transpeptidase activity. We further show that oxidative stress broadly interferes with IRES-dependent translation in gastric epithelial cells, affecting both cellular and viral IRES elements. The proposed working model (Figure 6) integrates these observations and suggests that redox-dependent inhibition of cap-independent translation of key survival and cell-cycle regulators, such as Survivin, may contribute to defective epithelial regeneration during chronic infection. Beyond bacterial pathogenesis, these findings underscore the importance of context-dependent regulation of cellular IRES-like elements and identify cap-independent translation as a potential target for therapeutic intervention in infection-associated diseases and cancer.

## 5. Conclusions

These data reveal a cap-independent activity within the Survivin 5′UTR, which is inhibited by *H. pylori* GGT. Such findings may help clarify how Survivin, along with other cellular proteins whose translation relies on cap-independent (IRES-mediated) mechanisms, is downregulated during *H. pylori* infection in the gastric epithelium.

## Data Availability

Any additional information required to reanalyze the data reported in this paper is available from the authors upon request.

## Supplementary Materials

The following supporting information can be downloaded at: https://www.mdpi.com/article/10.3390/biom16010164/s1, Figure S1: Original blots of Figure 1D; Figure S2: Original blots of Figure 1E; Figure S3: Original blots of Figure 5A.

## Abbreviations

The following abbreviations are used in this manuscript:

5′UTR	5′ untranslated region
poly(A)+	RNA polyadenylated
Cap+	Capped RNA
*H. pylori*	*Helicobacter pylori*
HCV	Hepatitis C virus
IRES	Internal ribosome entry site
GGT	gamma-glutamyl transpeptidase
HIV-1	Human Immunodeficiency Virus 1
ATO	Arsenic trioxide
ROS	Radical oxygen species
EMVC	Encephalomyocarditis virus
RLuc/FLuc	Renilla/Firefly luciferases
ITAF	IRES trans-acting factor
Surv 5′UTR_S_	Survivin 5′UTR short variant
Surv 5′UTR_L_	Survivin 5′UTR long variant
RRL	Rabbit reticulocyte lysate
MOI	Multiplicity of infection
