# Supplementary material for: The Cap-Independent Translation of Survivin 5′UTR and HIV-1 IRES Sequences Is Inhibited by Oxidative Stress Produced by H. pylori Gamma-Glutamyl Transpeptidase Activity
Source: Biomolecules. 2026 Jan 19;16(1):164. doi: 10.3390/biom16010164 (PMC12839084; doi:10.3390/biom16010164)
Supplement: Supplementary file 1 [file biomolecules-16-00164-s001.zip › biomolecules-4093502-supplementary.pdf]

The cap-independent translation of Survivin 5’UTR and HIV-1 IRES sequences is inhibited by oxidative stress produced by *H. pylori* gamma-glutamyl transpeptidase activity

Mariaignacia Rubilar [1], Nicolás Carrasco-Veliz [1], Maritza P. Garrido [2], María I. Silva [1]. Andrew F.G. Quest [3,4], María Fernanda González [3,4], Esteban Palacios [1], Joan Villena [5], Iván Montenegro [5] and Manuel Valenzuela-Valderrama [1*]

[1]Laboratorio de Carcinogénesis Molecular, Instituto de Investigación y Doctorados, Facultad de Ciencias de Medicina y Ciencias de la Salud, Universidad Central de Chile, Lord Cochrane 418, Santiago 8320000, Chile; manuel.valenzuela@ucentral.cl
[2]Laboratorio de Endocrinología y Biología de la Reproducción, Departamento de Obstetricia y Ginecología, Hospital Clínico Universidad de Chile, Avenida Santos Dumont 999, Independencia 8380456, Chile; mgarrido@hcuch.cl
[3]Centro de Estudios Avanzados en Enfermedades Crónicas (ACCDiS), Sergio Livingstone 1007, Independencia 8380492, Chile.
[4]Laboratorio de Comunicaciones Celulares, Centro de estudios en Ejercicio, Metabolismo y Cáncer (CEMC), Instituto de Ciencias Biomédicas (ICBM), Facultad de Medicina, Universidad de Chile, Independencia, Santiago 8380000, Chile; aquest@u.uchile.cl
[5]Centro de Investigaciones Biomédicas (CIB), Facultad de Medicina, Campus de la Salud, Universidad de Valparaíso, Angamos 655, Viña del Mar 2520000, Chile; juan.villena@uv.cl, ivan.montenegro@uv.cl
*Correspondence: M.VV., manuel.valenzuela@ucentral.cl; Tel.: +56-22-5851338

Figure S1:

Figure 1D. Original blots

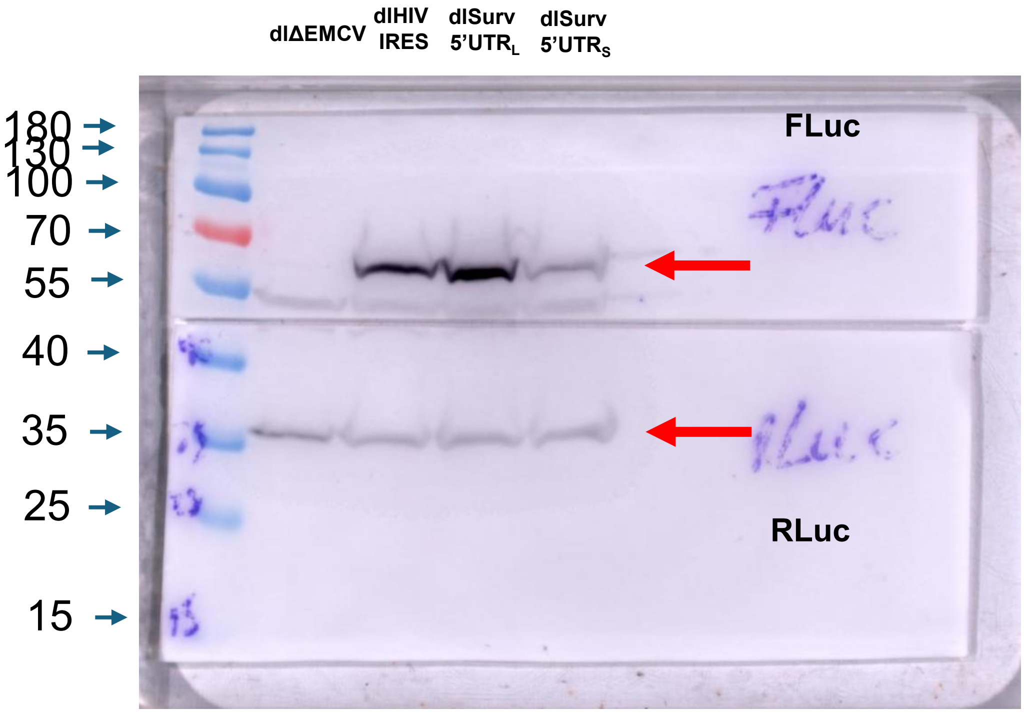

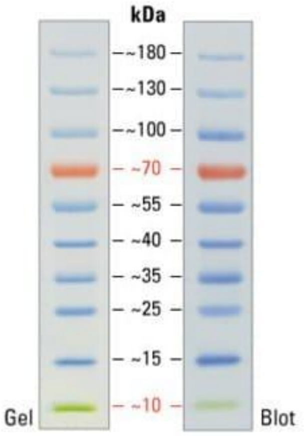

https://www.thermofisher.com/order/catalog/product/26616

Figure 1D.

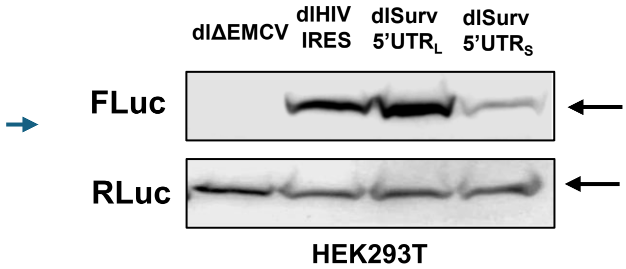

Figure S2: Figure 1E.

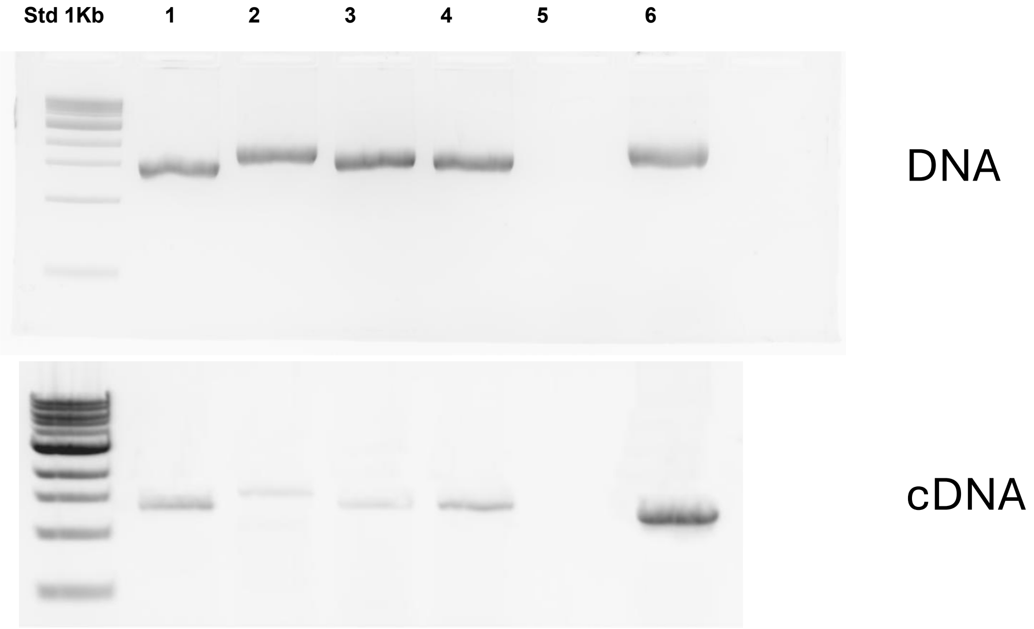

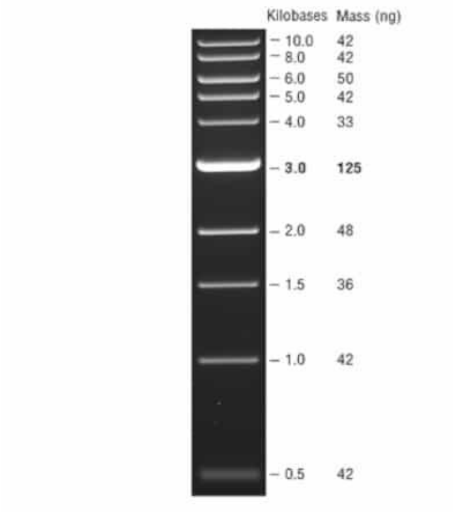

1kb NEB ladder

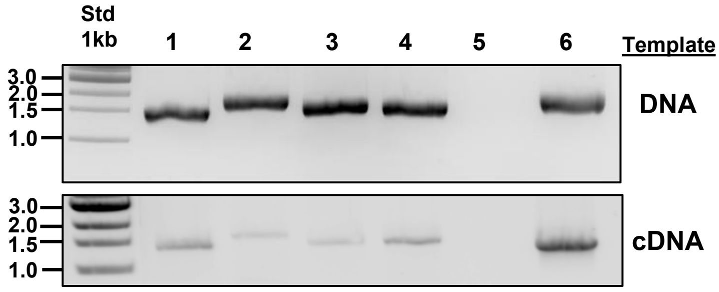

1. dl ΔEMCV
2. dl HIV IRES
3. dl Surv 5’UTR$_S$
4. dl Surv 5’UTR$_L$
5. NTC
6. Positive control (dl HIV IRES DNA)

Figure S3:

Figure 5A. Original blots

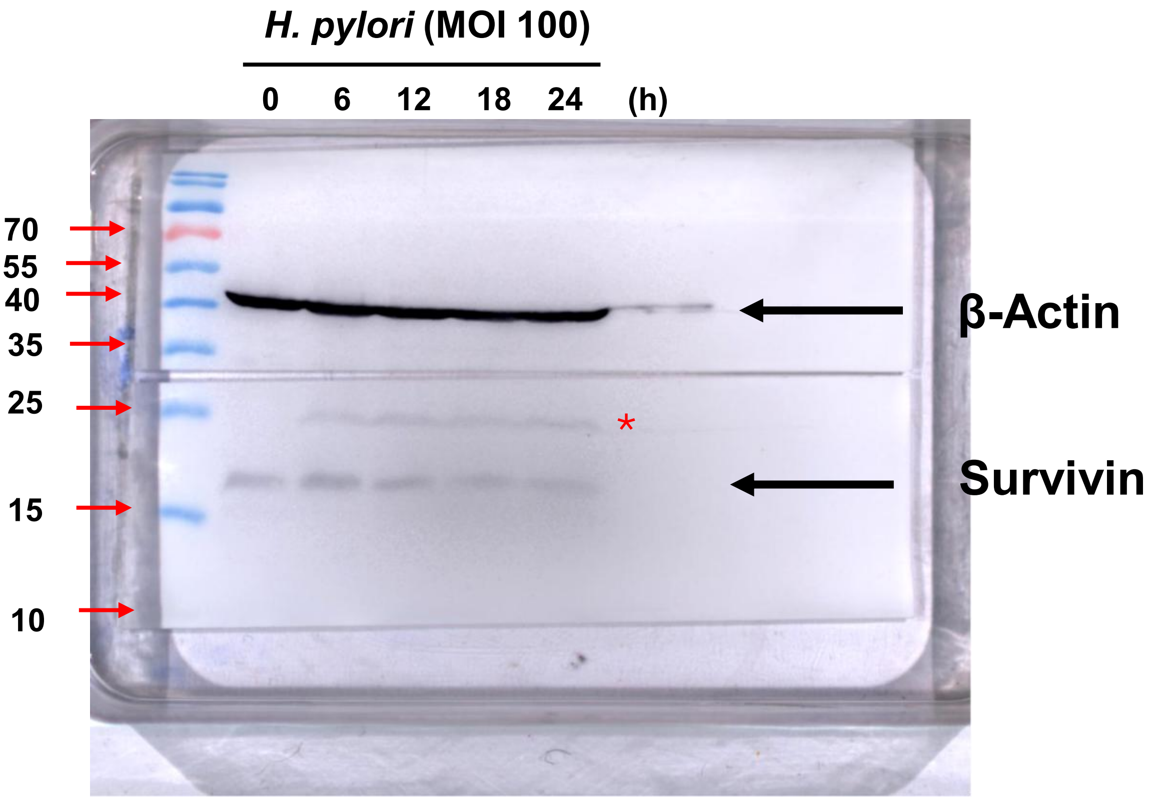

*The anti-survivin antibody recognizes an unidentified *Helicobacter pylori* protein

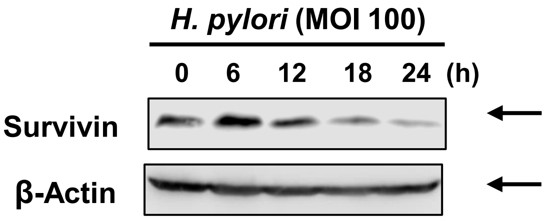

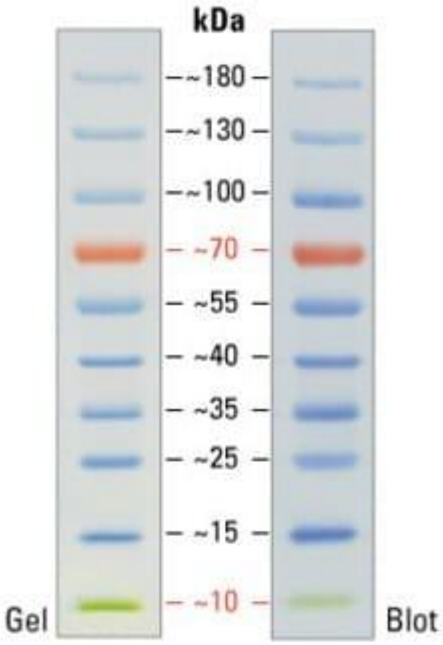

https://www.thermofisher.com/order/catalog/product/26616
